# Contribution of education level and dairy fat sources to serum cholesterol in Russian and Finnish Karelia: results from four cross-sectional risk factor surveys in 1992–2007

**DOI:** 10.1186/1471-2458-12-910

**Published:** 2012-10-26

**Authors:** Laura Paalanen, Ritva Prättälä, Tiina Laatikainen

**Affiliations:** 1 Department of Chronic Disease Prevention, National Institute for Health and Welfare (THL), P.O. Box 30, Helsinki, FI-00271, Finland; 2 Department of Lifestyle and Participation, National Institute for Health and Welfare (THL), P.O. Box 30, Helsinki, FI-00271, Finland; 3 The Institute of Public Health and Clinical Nutrition, University of Eastern Finland, Kuopio, Finland

**Keywords:** Russia, Finland, Education, Diet, Cholesterol, Saturated fat

## Abstract

**Background:**

Food habits vary by socio-economic group and geographic area. Data on socio-economic differences in food habits and in serum total cholesterol concentration from Russia are scarce. Our aim was to examine changes and educational differences in serum total cholesterol and in the consumption of major sources of saturated fat in two geographically neighbouring areas, Russian and Finnish Karelia, and to examine whether the foods associated with serum total cholesterol are different in the two areas.

**Methods:**

Data from cross-sectional risk factor surveys from years 1992, 1997, 2002 and 2007 in the district of Pitkäranta, the Republic of Karelia, Russia (*n* = 2672), and North Karelia, Finland (*n* = 5437), were used. The analyses included two phases. 1) To examine the differences in cholesterol by education, the means and 95% confidence intervals for education groups were calculated for each study year. 2) Multivariate linear regression analysis was employed to examine the role of butter in cooking, butter on bread, fat-containing milk and cheese in explaining serum total cholesterol. In these analyses, the data for all four study years were combined.

**Results:**

In Pitkäranta, serum total cholesterol fluctuated during the study period (1992–2007), whereas in North Karelia cholesterol levels declined consistently. No apparent differences in cholesterol levels by education were observed in Pitkäranta. In North Karelia, cholesterol was lower among subjects in the highest education tertile compared to the lowest education tertile in 1992 and 2002. In Pitkäranta, consumption of fat-containing milk was most strongly associated with cholesterol (β=0.19, 95% CI 0.10, 0.28) adjusted for sex, age, education and study year. In North Karelia, using butter in cooking (β=0.09, 95% CI 0.04, 0.15) and using butter on bread (β=0.09, 95% CI 0.02, 0.15) had a significant positive association with cholesterol.

**Conclusions:**

In the two geographically neighbouring areas, the key foods influencing serum cholesterol levels varied considerably. Assessment and regular monitoring of food habits are essential to plan nutrition education messages that are individually tailored for the target area and time.

## Background

Serum cholesterol is one of the major well-established risk factors for cardiovascular disease (CVD). Despite a steep decline in Finnish North Karelia during recent decades, CVD still accounts for a large part of premature morbidity and deaths [1-3]. In North Karelia, serum cholesterol concentrations were extremely high in the 1970s [4,5]. However, the average serum total cholesterol concentration among North Karelians has declined from 6.9 to 5.5 mmol/l in men and from 6.8 to 5.2 mmol/l in women from 1972 to 2007. The favourable decline in cholesterol was initiated by a comprehensive community-based intervention programme, ‘the North Karelia project’, launched in 1972, and supported by actions taken under the Finnish nutrition policy programmes [5-7].

On the other side of the Finnish-Russian border, in the district of Pitkäranta, the Republic of Karelia, cholesterol concentrations have tended to be lower than in North Karelia even if CVD is a common cause of death [8]. However, the serum cholesterol concentrations increased slightly from 5.2 to 5.3 mmol/l in men and from 5.3 to 5.5 mmol/l in women from 1992 to 2002 [9].

Serum cholesterol is greatly affected by diet, above all the quality of fat, in addition to other behavioural factors and genetics [10]. High intake of saturated fat elevates the cholesterol concentrations. The main sources of saturated fat in diet in most countries are dairy products and meat. In Finland, the intake of saturated fat has declined as margarine and skimmed milk have partly replaced butter on bread and high-fat milk [11]. The data on saturated fat intake in Russia are scarce, but there is evidence that at least the use of butter in cooking declined between 1994 and 2004 in the district of Pitkäranta in the Republic of Karelia [12].

The sources of saturated fat vary across socioeconomic status groups. In Finland, for example, the consumption of sausages is more typical among persons with a low education, whereas consumption of cheese is more common among persons with a higher educational level [13,14]. Similar socioeconomic patterns have been observed in other European countries as well [15]. Studies on socioeconomic differences in food consumption in Russia are scarce, but they point to weaker and partly opposite educational differences [12].

Our aim was to examine changes and educational differences in serum total cholesterol and in the consumption of major sources of saturated fat in diet from 1992 to 2007 in the district of Pitkäranta in the Republic of Karelia, Russia, and North Karelia, Finland. In addition, we wanted to examine whether different foods are associated with serum cholesterol in these two areas.

## Methods

### Study sites and population

The Republic of Karelia in Russia and the province of North Karelia in Finland are neighbouring areas with 296 km of common border. They partly shared a common history until the end of the Second World War, when parts of the formerly Finnish Karelia – including our study site, the district of Pitkäranta – were annexed to the Soviet Union. The Republic of Karelia is part of the Russian Federation. In the district of Pitkäranta, there are some 24 000 inhabitants, about half of whom live in the city of Pitkäranta. North Karelia is the easternmost province of Finland, with 167 000 inhabitants in 2007 [16].

The data were collected by cross-sectional risk factor surveys in 1992, 1997, 2002 and 2007. All surveys were conducted during the spring. The samples were stratified with each 10-year age group having an equal number of men and women. The survey methodology has been described in more detail earlier [17,18]. The methodology closely followed the WHO MONICA protocol [19]. The age range of subjects in this study was 25–64 years. The basic characteristics of the subjects are presented in Table 1. The study was approved by the internal review board of the National Public Health Institute, Finland (currently the National Institute for Health and Welfare, THL). Prevailing ethical instructions were applied in all studies. In Pitkäranta, Russia, local ethical practice was applied. Regarding data collection in North Karelia, Finland, the studies were conducted according to the guidelines laid down in the Declaration of Helsinki. All procedures involving human subjects were approved by the prevailing ethics committees, the latest being the Coordinating Ethics Committee, Hospital District of Helsinki and Uusimaa. Since 2002, written informed consent was obtained from all subjects.

**Table 1:** **Number of participants**, **mean cholesterol concentration and prevalence of milk**, **butter and cheese consumption with 95**% **confidence intervals in Pitkäranta**, **the Republic of Karelia and North Karelia**, **Finland**, **1992**–**2007 **^**a**^

	**1992**	**1997**	**2002**	**2007**
**Pitkäranta**, ***n*** = **2672**				
Number of participants	835	749	605	483
Total cholesterol, mmol/L (mean)	5.26 (5.19–5.33)	**5**.**10** (**5**.**02**–**5**.**18**)	**5**.**42** (**5**.**33**–**5**.**51**)	5.43 (5.29–5.56)
Butter in cooking (%)	65 (62–69)	**20** (**17**–**23**)	**11** (**9**–**14**)	**11** (**9**–**14**)
Butter on bread (%)	76 (73–79)	**39** (**35**–**42**)	**47** (**43**–**51**)	**57** (**53**–**62**)
Consumption of fat-containing milk (%)	78 (75–80)	**62** (**59**–**66**)	**69** (**65**–**73**)	**61** (**57**–**66**)
Daily consumption of fatty cheese (%)	3 (2–4)	**13** (**11**–**16**)	**21** (**18**–**24**)	**40** (**35**–**44**)
**North Karelia**, ***n*** = **5437**				
Number of participants	1476	1463	1426	1072
Total cholesterol, mmol/L (mean)	5.72 (5.66–5.78)	**5**.**56** (**5**.**50**–**5**.**61**)	**5**.**56** (**5**.**51**–**5**.**62**)	**5**.**28** (**5**.**21**–**5**.**34**)
Butter in cooking (%)	56 (53–58)	**43** (**40**–**46**)	**37** (**34**–**39**)	**31** (**28**–**34**)
Butter on bread (%)	32 (30–34)	**22** (**20**–**25**)	**24** (**22**–**26**)	**22** (**20**–**25**)
Consumption of fat-containing milk (%)	67 (65–69)	**47** (**44**–**49**)	**42** (**40**–**45**)	**36** (**33**–**39**)
Daily consumption of fatty cheese (%)	50 (47–52)	53 (50–55)	**44** (**41**–**47**)	**30** (**27**–**33**)
Daily consumption of low-fat cheese (%)	16 (14–18)	**22** (**20**–**24**)	**38** (**35**–**40**)	**42** (**39**–**45**)

^a^Statistically significant figures compared to 1992 are written in bold.

### Measurements

Data on socio-economic status and food consumption were derived from self-administered questionnaires. Education was chosen to indicate socio-economic position. Education was measured as the total number of years of education. The years of education were divided into tertiles (high, intermediate and low) within each ten-year birth cohort in the two areas, separately for men and women. This relative education variable was used because the educational systems differ between Finland and Russia, and it is not possible to define simpler categories that would aptly describe the subjects’ educational level in a comparable manner. In addition, the data included persons born between the years 1928 and 1982 and the distributions of years of education have changed over time.

In North Karelia, the venous blood samples were centrifuged at the survey site in 1992, 1997 and 2002, and the sera were mailed daily for cholesterol measurements to the laboratory of the National Public Health Institute, Helsinki, Finland. The laboratory is accredited and standardised against national and international reference laboratories. In 2007, the sera were frozen immediately after separation and transferred in dry ice to the laboratory once a week for analyses. In Pitkäranta, the methodology was as identical as possible, and the analyses were made from fresh serum samples in 1992–2002. The samples were transferred once or twice a week to the laboratory of the National Public Health Institute, Helsinki, Finland. In 2007, the sera were frozen and transferred about every other week to the laboratory. The serum total cholesterol was analysed using an enzymatic method (CHOD-PAP, Boehringer-Mannheim, Monotest). More information on the cholesterol measurement methods can be found in a previous publication [20].

To represent important sources of saturated fat we chose 1) use of butter in cooking, 2) use of butter on bread, 3) consumption of fat-containing milk, and 4) daily consumption of fatty cheese. From North Karelia, trends for daily consumption of low-fat cheese are also presented. Low-fat cheese was not available in Pitkäranta during the study period. Categories from multiple-choice questions were combined to construct the dichotomous outcome variables ‘butter in cooking’, ‘butter on bread’ and ‘consumption of milk containing fat’. The format of the questions was ‘What kind of fat is usually used in your home for food preparation / on bread?’ Hard margarine and mixtures of butter and oil consisting mainly of saturated fat were categorised as ‘butter’. The reference category included other fats like vegetable oil and soft margarine and using no fat in cooking or on bread. Regarding milk consumption, skimmed milk was not available in Pitkäranta during the study period. Thus, in Pitkäranta, ‘consumption of fat-containing milk’ included all persons who drank milk, and those who did not were categorised in the reference category. In North Karelia, subjects drinking skimmed milk and subjects that did not drink any milk belonged to the reference category. Respondents were asked about their consumption of cheese in a food frequency section, which is described in more detail in our previous publication [21]. In North Karelia, the format of the food frequency section was revised in 2007, but categories comparable with other study years could be constructed from the new food frequency section.

### Statistical methods

Data for the two areas, the district of Pitkäranta and North Karelia, were analysed separately. Because the preliminary analyses showed that the results were very similar for men and women in both areas, we decided to analyse the data for men and women combined adjusting for sex.

The statistical analyses included two phases.

1) To examine the differences in cholesterol by education, the means and 95% confidence intervals (CI) for education groups were calculated for each study year. The prevalences of food consumption groups with 95% CI were also calculated. Since the samples were stratified in 10-year age groups, the point estimates do not accurately describe the populations. However, the use of an identical sampling method each year in both areas makes it possible to describe trends and compare the figures between the two areas.

2) The role of selected foods in explaining serum total cholesterol was examined using multivariate linear regression analysis. In these analyses, the data for all four study years were combined. In the analyses, three models were employed. Model 1 included adjustments for sex and age. Model 2 added the study year and Model 3 the study year and education. In the adjustments, study year was used as a continuous variable, and the values were coded as 1–4. Only one food variable was included in the analyses at a time.

Finally, to check whether the effect of the selected foods varied depending on education level, the terms for interaction between the food variables and education were tested.

Analyses were conducted using the STATA statistical software package version 11.2 (StataCorp., College Station, TX, USA).

## Results

### Changes in serum cholesterol levels and dairy fat consumption from 1992 to 2007

**Figure 1:**
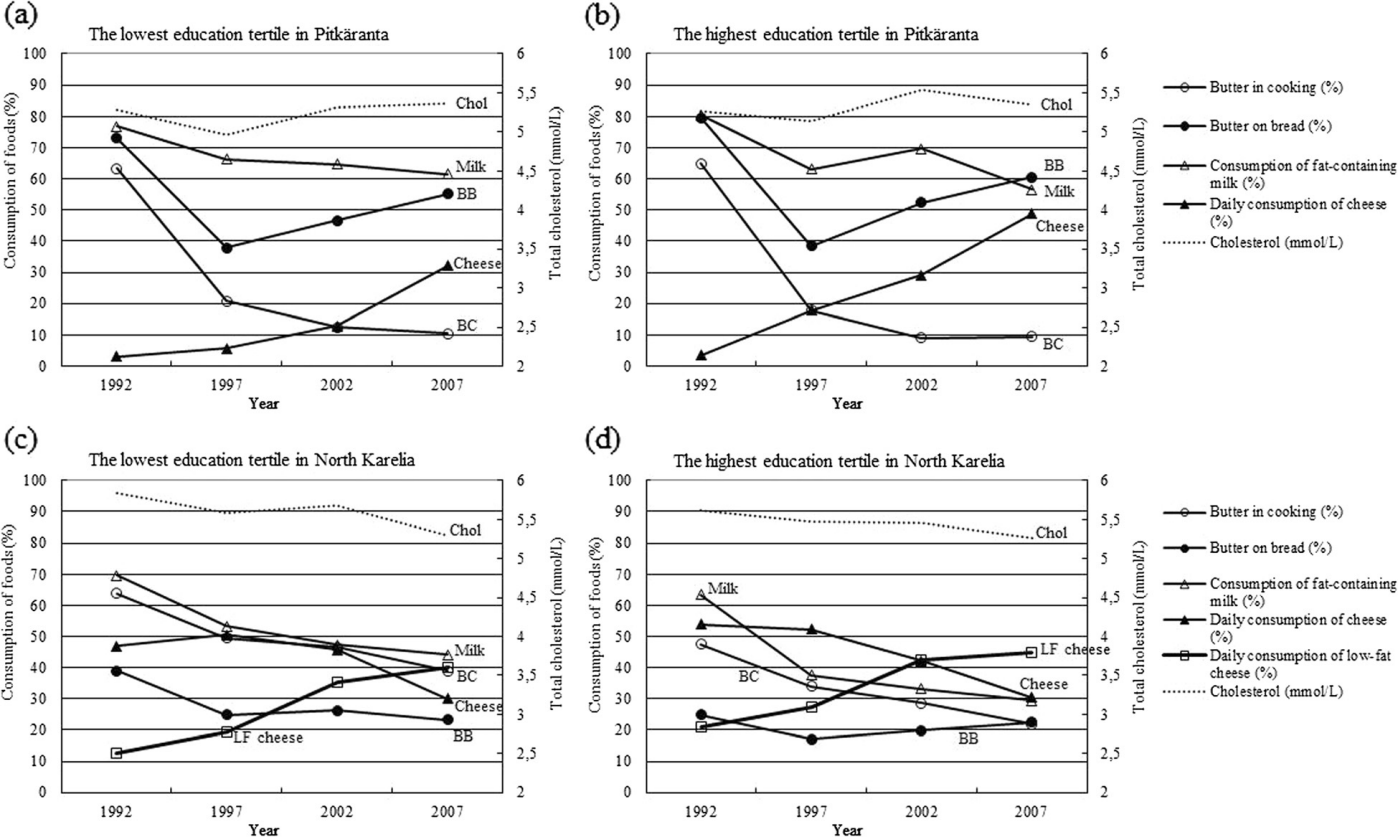
**Trends in serum cholesterol levels and consumption of milk**, **butter and cheese.** Data shown for the lowest and highest education tertiles in Pitkäranta, the Republic of Karelia, and North Karelia, Finland, 1992–2007. BC = butter in cooking; BB = butter on bread; Milk = consumption of fat-containing milk; Cheese = daily consumption of cheese; LF Cheese = daily consumption of low-fat cheese; Chol = cholesterol.

The serum cholesterol levels in Pitkäranta fluctuated during the study period (Table 1, Figure 1). Cholesterol levels decreased from 1992 to 1997, but thereafter, cholesterol has been on the increase in Pitkäranta. In North Karelia, cholesterol levels decreased significantly from 1992 to 2007 (Table 1, Figure 1). The decrease was significant in all education groups (data not shown).

On the whole, in both areas the food habits selected as indicators for saturated fat became less common during the study period, with the exception of a marked increase in the consumption of fatty cheese in Pitkäranta (Table 1, Figure 1). In Pitkäranta, in addition, the use of butter on bread decreased from 1992 to 1997, but increased steadily thereafter. In North Karelia, all food habits indicating saturated fat intake became less common. Simultaneously, in North Karelia, daily consumption of low-fat cheese became more common during the study years.

### Serum cholesterol levels and dairy fat consumption by education

In Pitkäranta, serum total cholesterol concentrations did not differ by education (Figure 1, values not shown). In North Karelia, mean serum total cholesterol was lower in the highest education group (5.61 mmol/l, 95% CI 5.52, 5.71 mmol/l) compared to the lowest education group (5.84 mmol/l, 95% CI 5.74, 5.94 mmol/l) in the beginning of the study period in 1992. The same was true in 2002 (mean total cholesterol 5.45 mmol/l, 95% CI 5.37, 5.54 mmol/l in the highest education group, 5.68 mmol/l, 95% CI 5.58, 5.78 mmol/l in the lowest education group). In 1997, the educational difference did not reach statistical significance. From 2002 to 2007, the educational difference in cholesterol levelled off in North Karelia.

Regarding food consumption, daily consumption of fatty cheese tended to be more common among subjects in the highest education group compared to the lowest education group in Pitkäranta (Figure 1, values not shown). The difference was statistically significant since 1997. In North Karelia, the subjects who most often used butter in cooking across the study years were those with low education (Figure 1, values not shown). The difference was statistically significant. In North Karelia, parallel educational differences were observed also for using butter on bread in 1992 and 1997, and for drinking fat-containing milk in 1997, 2002 and 2007. In addition, in 1992 the daily consumption of low-fat cheese was more common among North Karelians with high education compared to subjects with a lower education, but not in later studies.

### Foods associated with serum cholesterol

In Pitkäranta, consumption of fat-containing milk was most strongly associated with serum cholesterol in our study (Table 2). Adding study year to the analyses (Models 2 and 3) strengthened the association of fat-containing milk and cholesterol compared to Model 1, adjusting for sex and age only. In addition, using butter on bread was significantly associated with cholesterol in Pitkäranta when adjusting for sex, age and study year (Table 2, Model 2); however, it fell slightly short of significance in the two other models.

**Table 2:** **Food habits associated with serum cholesterol concentration from multivariate linear regression analysis **^**a**,**b**^

	**Model 1**^**c**^		**Model 2**^**d**^		**Model 3**^**e**^	
	**β**	**95%CI**	**β**	**95%CI**	**β**	**95%CI**
**Pitkäranta**						
Butter in cooking	0.01	(−0.08, 0.10)	0.08	(−0.01, 0.18)	0.08	(−0.02, 0.18)
Butter on bread	0.07	(−0.02, 0.15)	**0**.**08**	(**0**.**001**, **0**.**17**)	0.08	(−0.004, 0.16)
Consumption of fat-containing milk	**0**.**17**	(**0**.**08**, **0**.**26**)	**0**.**19**	(**0**.**10**, **0**.**28**)	**0**.**19**	(**0**.**10**, **0**.**28**)
Daily consumption of cheese	0.01	(−0.10, 0.13)	−0.04	(−0.16, 0.07)	−0.06	(−0.18, 0.06)
**North Karelia**						
Butter in cooking	**0**.**16**	(**0**.**10**, **0**.**21**)	**0**.**11**	(**0**.**05**, **0**.**17**)	**0**.**09**	(**0**.**04**, **0**.**15**)
Butter on bread	**0**.**13**	(**0**.**06**, **0**.**19**)	**0**.**11**	(**0**.**04**, **0**.**17**)	**0**.**09**	(**0**.**02**, **0**.**15**)
Consumption of fat-containing milk	**0**.**09**	(**0**.**04**, **0**.**15**)	0.03	(−0.03, 0.09)	−0.02	(−0.04, 0.07)
Daily consumption of cheese	−0.002	(−0.06, 0.06)	−0.04	(−0.10, 0.02)	−0.04	(−0.10, 0.02)

^a^β coefficients and 95% confidence intervals. Statistically significant associations are written in bold.

^b^Data for men and women and for years 1992, 1997, 2002 and 2007 were combined. In the adjustments, age and study year were included as continuous variables (values for study year were set as 1–4).

^c^Model 1 = adjusted for sex and age.

^d^Model 2 = adjusted for sex, age and study year.

^e^Model 3 = adjusted for sex, age, study year and education.

In North Karelia, on the contrary, using butter in cooking and using butter on bread were most strongly associated with serum cholesterol levels (Table 2) in our study.

### Interactions between dairy fat sources and education

]No significant interactions between selected dairy fat sources and education in predicting serum cholesterol were found in Pitkäranta (data not shown). In North Karelia, daily consumption of cheese had a statistically significant interaction with education in predicting cholesterol (p=0.03).

## Discussion

We examined trends and educational differences in serum total cholesterol and in the consumption of major sources of saturated fat in diet from 1992 to 2007 in the district of Pitkäranta in the Republic of Karelia, Russia, and North Karelia, Finland. We also examined which saturated fat sources were associated with serum cholesterol in the two areas. Our study period encompasses the years of major changes in the political and economical system in Russia after the collapse of the Soviet Union in 1991.

During the study period, serum cholesterol levels fluctuated in Pitkäranta. No apparent educational differences were seen. Dramatic changes in food habits selected as indicators of saturated fat intake occurred in Pitkäranta; using butter in cooking tumbled in all education groups, whereas consuming fatty cheese on a daily basis became substantially more common, even more so in the high education group than among subjects with a low educational level. In North Karelia, the changes in cholesterol levels were in line with the changes in food habits. As the consumption of foods with saturated fat decreased, so did the cholesterol levels in the population. In addition, as the educational differences in food habits narrowed, so did the educational difference in cholesterol levels. Parallel changes have been observed in Lithuania, a Baltic country that was part of the Soviet Union until 1991, where using butter on bread halved and using vegetable oil in cooking increased six fold from 1993 to 2007 [22]. In Lithuania, the changes in serum total cholesterol were in line with the favourable changes in diet; serum total cholesterol fell by about 0.50 mmol/l in 1993–2007. Consumption of cheese was not reported in this study.

The sources of saturated fat in diet that were associated with serum cholesterol levels were quite different in the two areas, namely the district of Pitkäranta in the Republic of Karelia, Russia, and North Karelia, Finland. In Pitkäranta, drinking fat-containing milk had the strongest association with serum cholesterol in our study. In North Karelia, Finland, on the other hand, using butter in cooking and using butter on bread were most strongly associated with cholesterol.

In Pitkäranta, drinking fat-containing milk may reflect other food habits that were not included in our study, e.g. frequent use of fatty meat or smetana (Russian sour cream), though it emerged as the single significant predictor of cholesterol in our study. In North Karelia, drinking fat-containing milk did not seem to be associated with cholesterol. However, in our analyses, ’drinking fat-containing milk’ included all milk types that contain some fat, and it is likely that ‘fat-containing milk’ had on average a lower fat content in the data from North Karelia compared to the data from Pitkäranta. This might partly explain the weaker association between milk and cholesterol in North Karelia. Unlike in North Karelia, low-fat milk was not readily available in Pitkäranta over the study years. Regarding the data from North Karelia, milk that contains only 1% fat was also included in the category ‘fat-containing milk’, even though Finnish nutrition recommendations recommend it together with skimmed milk.

In Pitkäranta, consumption of cheese was more common among subjects with higher educational level. Similar differences have been observed earlier in Finland as well [15], but in our study, which only included North Karelia in eastern Finland, the educational differences in fatty cheese consumption did not reach statistical significance. Daily consumption of low-fat cheese was more common among subjects with a high education in 1992. It seems that in Finland, persons with a higher education may have been the first to shift from fatty cheese to low-fat cheese, but the educational differences have levelled off with time.

Despite their common history before the Second World War, the district of Pitkäranta in the Republic of Karelia, Russia, and North Karelia in Finland have been economically and politically distinct from each other since. Our study in these two distinct areas gives rise to several methodological questions. One such issue is how feasible is it to use questionnaires that are basically similar in different settings, even if the questions and multiple-choice options have been modified to suit local circumstances. In many cases, the questionnaires have originally been designed for one target population, in our case Finnish citizens. For example, in Finland, it is very common to spread butter or margarine on bread. However, this is not the case in Russia, where bread is often eaten without spread and the important sources of fat are likely to be something else.

Our study only included dairy fat sources as indicators of saturated fat intake. There are, however, other important sources of saturated fat like meat and meat products as well [23]. A self-administered questionnaire with only a few food-related questions cannot include all important sources of saturated fats. Furthermore, it cannot measure the share of the total fat intake accounted for by saturated fat.

The situation with cardiovascular disease has been very different between North Karelia, Finland, and the Republic of Karelia, Russia. North Karelia started in the 1970’s with very high CVD rates and very high average serum cholesterol level and has had a fairly steady decline thereafter. On the other hand, Russia had initially much lower CVD rates, but during the last few decades marked increase. The initially lower level of serum cholesterol in Russia can obviously be explained by the low availability and accessibility of dairy fat products in Russia. The later increase indicates the changing economic situation, while in Finland active health promotion has been behind the marked reduction in the consumption of dairy fat products and CVD rates.

## Conclusions

In conclusion, in Pitkäranta in the Republic of Karelia, Russia, drinking fat-containing milk and in North Karelia, Finland, using butter in cooking and on bread were most strongly associated with cholesterol levels. Educational differences were more evident in North Karelia than in Pitkäranta. Our findings emphasise the importance of regular population surveys on food habits as a knowledge base for nutrition policies and education. As the sources of saturated fats and other nutrients vary between populations and change over time, food-related questions in health surveys and food-based dietary guidelines should always be tailored and updated according to the food habits of the target population.

## Competing interests

The authors declare that they have no competing interests.

## Authors’ contributions

TL was involved in the data collection. LP, RP and TL planned the study objectives of this paper. LP conducted the statistical analyses and drafted the manuscript. RP and TL were involved in interpretation of data and critically revised the manuscript written by LP. All authors read and approved the final manuscript.

## Pre-publication history

The pre-publication history for this paper can be accessed here:

http://www.biomedcentral.com/1471-2458/12/910/prepub
